# Canine Mammary Cancer: State of the Art and Future Perspectives

**DOI:** 10.3390/ani13193147

**Published:** 2023-10-09

**Authors:** Eliza Vazquez, Yulia Lipovka, Alejandro Cervantes-Arias, Adriana Garibay-Escobar, Michelle M. Haby, Felisbina Luisa Queiroga, Carlos Velazquez

**Affiliations:** 1 Department of Chemistry-Biology, University of Sonora, Blvd. Luis Encinas y Rosales s/n, Hermosillo 83000, Mexico; eliza.vazquez@unison.mx (E.V.); yulia.lipovka@unison.mx (Y.L.); adriana.garibay@unison.mx (A.G.-E.); haby@unimelb.edu.au (M.M.H.); 2 Department of Small Animal Medicine and Surgery, Small Animal Teaching Hospital, The National University of Mexico (UNAM), Ciudad Universitaria, Investigación Científica 3000, Coyoacán, Mexico City 04360, Mexico; a.cervantes@unam.mx; 3 CECAV—Animal and Veterinary Research Center, University of Trás-os-Montes and Alto Douro, 5001-801 Vila Real, Portugal

**Keywords:** mammary cancer, canine, molecular classification, diagnosis, treatment

## Abstract

**Simple Summary:**

Mammary tumors are the most frequent neoplasia in female dogs. They develop spontaneous cancer and share several biological, clinical, pathological and molecular characteristics with cancer diagnosed in humans. Mammary cancer is also one of the leading causes of death in both species. This review provides a detailed description of the histological, molecular and clinical aspects of mammary cancer in canines; it discusses risk factors and currently available diagnostic and treatment options, as well as remaining challenges and unanswered questions.

**Abstract:**

Mammary cancer is the most frequently diagnosed neoplasia in women and non-spayed female dogs and is one of the leading causes of death in both species. Canines develop spontaneous mammary tumors that share a significant number of biological, clinical, pathological and molecular characteristics with human breast cancers. This review provides a detailed description of the histological, molecular and clinical aspects of mammary cancer in canines; it discusses risk factors and currently available diagnostic and treatment options, as well as remaining challenges and unanswered questions. The incidence of mammary tumors is highly variable and is impacted by biological, pathological, cultural and socioeconomic factors, including hormonal status, breed, advanced age, obesity and diet. Diagnosis is mainly based on histopathology, although several efforts have been made to establish a molecular classification of canine mammary tumors to widen the spectrum of treatment options, which today rely heavily on surgical removal of tumors. Lastly, standardization of clinical study protocols, development of canine-specific biological tools, establishment of adequate dog-specific disease biomarkers and identification of targets for the development of new therapies that could improve survival and have less adverse effects than chemotherapy are among the remaining challenges.

## 1. Introduction

Cancer is a heterogeneous group of diseases characterized by an uncontrolled proliferation of abnormal cells that can spread to the surrounding tissues. It is one of the most common causes of death in humans and dogs. In humans, around 10 million cancer-related deaths are reported and 19.3 million new cases are diagnosed annually, while in dogs, 4 million new cancer cases are diagnosed every year [1,2,3]. Cancer is the first cause of death in dogs over 10 years of age, with 50% of them developing this disease and one in four dying because of cancer [4]. Canines develop spontaneous cancer and share several biological, clinical, pathological and molecular features with humans [5,6,7]. Mammary tumors, affecting numerous mammal species, are the most common neoplasia diagnosed in female dogs and women, and they are considered to be a major problem in public health [5]. Gaining insight into the presentation and progression of breast cancer across different species will help us to better understand the pathogenesis of this complex disease [8].

Apart from being sentinels of the environment and human lifestyle, dogs with tumors are considered to be study models for the development of new drugs or therapies and for clinical trials [6,8,9,10]. The aims of this review are to describe the state of the art of canine mammary cancer and provide an update on current knowledge of its epidemiology, clinical and molecular aspects, as well as diagnosis and treatment options. Lastly, challenges and areas of opportunity are identified to help improve the outcomes of canines with mammary tumors.

## 2. Canine Mammary Tumors

Canine mammary tumors are an overly frequent condition in comparison to other types of cancer; they represent 50–70% of all neoplasia diagnosed in non-spayed female dogs, mainly affecting canines over 7 years of age. They appear as nodules of different sizes and are usually well-defined. The treatment regimen and prognosis of the patient can be established according to the physical characteristics, location, histological and molecular classification of the tumor. The incidence of canine mammary tumors varies depending on the geographic location of the study, and it is also affected by the age, hormonal exposition, breed and molecular features of the female dog, among other factors. In the next sections, a detailed description of the epidemiological, histological and molecular features of canine mammary tumors is presented, and factors affecting incidence, treatment and prognosis are discussed.

### 2.1. Epidemiological Features

#### 2.1.1. Incidence and Distribution

Information on the incidence of canine mammary tumors worldwide is very limited and only available for a few countries in Europe and North America. This review, therefore, focuses on the data available in these countries. In Table 1, the incidence rate expressed per 100,000 and 10,000 dogs per year is shown, and in the text below there is additional epidemiological information. As seen in Table 1, the incidence of canine mammary tumors varies in every country and over time. This variation can be attributed to several factors, with spaying culture being one of them. Spaying is usually performed as a canine population control measure. However, castration at early ages also prevents mammary tumor development in the female dog [11] since estrogens and progesterone produced by the ovaries are mitogens for the mammary epithelium and can stimulate duct and lobe proliferation and growth [12].

In Europe, the highest incidence rate of mammary carcinoma has been identified in the south, where neutering is not performed routinely [19,20]. In Sweden, a study performed on 80,000 female dogs, insured for veterinary medical attention and with life insurance, determined an incidence rate for mammary tumors of 111 cases for every 10,000 dogs every year, from 1995 to 2002, which is even higher than the numbers reported above [15]. In Denmark, an analysis of 1878 neoplasms from the veterinarian cancer register in the years 2005 to 2008 found that 500 corresponded to mammary tumors. Of all female reproductive system neoplasm cases reported (including mammary glands), 44% were malignant and 42% benign [21]. In the Canary Archipelago, researchers analyzed the data from 7362 canine mammary tumors in the period 2003–2020. They found that 5.32% were mixed tumors, 7.59% benign and 87.08% malignant, with a higher proportion of malignant tumors than found in Denmark or Mexico [5,15,22].

In Portugal, data were collected between 2019 and 2020 from the registries of the Animal Cancer Register; 7334 canine cases were analyzed and 1587 cases presented mammary tumors of which 60.9% were benign and 39.1% were malignant, with a lower proportion of malignant tumors than the reports from Denmark, the Canary Archipelago and Mexico [5,21,22,23].

In 1970, in California, in the United States of America, the incidence rate of mammary tumors was 145 cases for every 100,000 dogs every year [18]. Since then, the incidence of mammary tumors in the USA and Western Europe has decreased considerably and is lower compared to other countries, due to the wider use of the neutering procedure performed at an early age in dogs [24].

In other countries, including India and Mexico, there are few publications on the incidence of mammary tumors, and the majority are based on small sample sizes analyzed in universities, due to a lack of official canine population registries. Therefore, the samples selected for the study, are unlikely to be representative of the country or region. One of these studies was conducted at the veterinarian pathology department of the University of Ludhiana, in India, using 169 biopsy samples collected between December 2008 and April 2010. It was found that 109 were malignant neoplasms and 51 of them were mammary carcinoma; the incidence of mammary tumors was reported at 46.79% [25].

In a study conducted in the veterinarian pathology department of the National Autonomous University of Mexico (UNAM) from 2002 to 2012, 1917 biopsy samples of canine mammary lesions were analyzed, and the annual incidence rate of mammary carcinoma was determined to be 16.8%. Of these, 47.7% were benign and 47.5% were malignant tumors. The remaining 4.7% were diagnosed as non-neoplastic lesions, including hyperplasia, dysplasia or inflammation [5].

Regarding the most frequently diagnosed malignant mammary tumors, the statistics vary from country to country. Moreover, most of the studies reporting the incidence of canine mammary tumors were not performed during the same period of time; therefore, a direct comparison cannot be made. A study performed in Veneto, Italy, identified complex carcinoma and simple carcinoma as the most common types [14]. In Mexico, simple carcinoma, complex carcinoma and mixed carcinomas are the most prevalent [26]. In India, the most frequent malignant mammary tumors were reported as papillary adenocarcinoma, mixed carcinoma and solid carcinoma [27]. In Korea, the most frequently diagnosed malignant mammary tumors are mixed carcinoma, complex carcinoma and tubulopapillary carcinoma [28]. There is a need for additional studies in other countries, and there is a need to establish a link between the incidence of different cancer types, survival rates and tumor progression in canines.

As we have shown, information regarding canine mammary tumors is still very limited on a global level, mainly due to the lack of censuses, registers and studies that can determine the exact canine population in each region. In addition, the majority of the studies lack a population-oriented approach, and, therefore, the results often cannot be extrapolated to the entire population [29]. For example, studies conducted in Sweden, Denmark and Italy are based on data found in databases of insurance companies, health institutions, animal tumor registries and veterinary hospitals. While these sources allow the identification of epidemiologic characteristics like the incidence, breed predisposition, age of presentation and histologic types, among others, the results obtained only represent canines that are insured or go to veterinary centers or hospitals and cannot be generalized to the relatively large group of dogs that do not have access to these services. Thus, further studies that include other canine populations in different countries are required to determine whether these statistics apply to the overall dog population and better identify risk factors and prognosis.

#### 2.1.2. Etiology and Risk Factors

**Figure 1:**
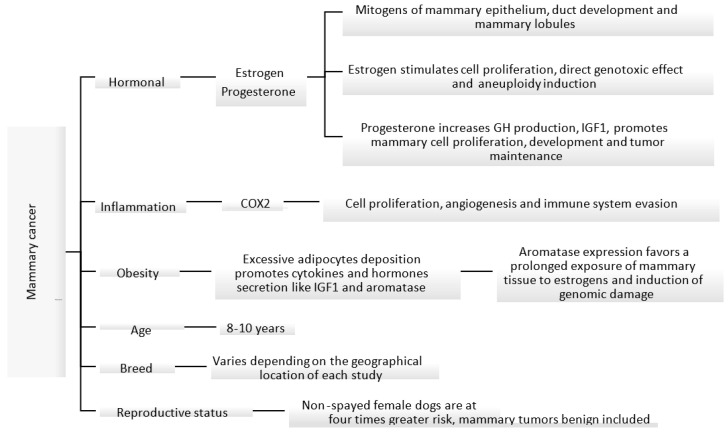
Known mechanisms and factors involved in the induction of canine mammary cancer [31,35,36,37,38,39].

The etiology of canine mammary carcinoma is not fully understood; however, similarly to women’s breast cancer, its development is impacted by hormonal, genetic, nutritional and environmental factors [30,31,32,33]. Some of these factors are shown in Figure 1. Most malignant mammary tumors develop in middle-aged (5–7 years of age) and elderly (7–8 years of age) female dogs, with the median age of presentation ranging between 8 and 10 years [5,24,34]. In addition, the risk of developing mammary tumors increases with the delay in spaying [5,31,35]. Non-spayed female dogs are at four times greater risk of developing mammary tumors, compared to those spayed before two years of age [35]. Another study found that the incidence of canine mammary tumors in dogs spayed before the first heat was 0.05%, and it increased as the number of heats progressed, being 8% and 26% after the first and second heat, respectively. In females spayed after their third heat, the risk of developing a mammary tumor was similar to that of a non-spayed female dog [18,35].

In female dogs and women, ovarian steroids stimulate the normal growth of mammary tissue under physiologic conditions. However, the proliferative effect in the epithelium can create the perfect environment for neoplastic proliferation. Ovarian hormones, mainly estrogens and progesterone, play an important role in the development of mammary tumors [31,40]. During the luteal phase, mammary tissue is exposed to high levels of progesterone, which could lead to growth hormone (GH) up-regulation. This hormone is believed to stimulate the mammary stem cells as the first step of carcinogenesis [36]. GH increases insulin growing factor I (IGF-I) levels, which in turn stimulates the proliferation of mammary cells and acts as a local growing factor, promoting tumor development and maintenance [30,31,36]. Pseudopregnancy has no relationship with the development of mammary tumors [24,41]; however, the use of progesterone as a contraceptive can induce the development of benign mammary tumors in canines. Synthetic progestins, like medroxyprogesterone acetate, promote similar effects to endogenous progesterone in the mammary glands [42,43].

Estrogens can promote a pro-carcinogenic effect through inhibition of apoptosis and induce genetic/epigenetic changes that modulate the expression of genes involved in the regulation of cell proliferation and differentiation [40]. Estrogen-induced cell proliferation increases the incidence of genetic alterations. In addition, metabolites derived from the oxidative metabolism of estradiol can cause direct genotoxic effects [37,38]. High levels of steroidal hormones have been identified in serum and in mammary tissue in female dogs with malignant tumors in comparison to those with benign tumors, suggesting that steroidal hormones act as local growing factors, stimulating the proliferation of cells [31]. In addition, estrogen and progesterone receptors are also present in both benign and malignant lesions [30]. In female dogs, obesity is a risk factor for the early development of high-grade mammary tumors and is also associated with shorter survival [39,44]. Young overweight dogs fed with a diet high in red meats are at a higher risk of developing mammary tumors and dysplasia [45]. Conversely, the risk of mammary carcinoma decreases greatly in spayed juvenile (9 to 12 months of age) female dogs with a thin body conformation [46]. Dogs fed with homemade food have a higher body condition score compared to those fed with a commercial diet; however, there does not seem to be a relationship between diet and cancer survival rates [44]. Obesity and a high-fat diet have also been associated with an increased risk of breast cancer in women. Women with breast cancer and obesity experience increased tumor proliferation, risk of recurrence and decreased survival [47].

At the cellular level, obesity causes inflammation of the adipose tissue with activation of macrophages that produce inflammatory mediators such as tumor necrosis factor α and interleukin 6 and other substances like leptin, adiponectin, resistin and aromatase. This can lead to increased cell proliferation, inhibit apoptosis and induce angiogenesis [39]. In addition, adipose tissue and high cholesterol levels can be an important source of steroidal hormones including estrogens, progesterone and androgens. Peripheral aromatization of androgens to estrogens can lead to prolonged exposure of mammary tissue to estrogens [48]. In fact, one study found that aromatase expression increased in overweight female dogs with mammary carcinoma and therefore might impact its progression through hormonal receptor signaling [39]. Another study evaluated adiponectin expression in canine mammary tumors and found 49% (36/76) of the patient tumors to be positive; however, no correlation was found with tumor size, histotype, grade or presence of lymphatic invasion [44]. Adiponectin, a protein secreted by the adipose tissue, is negatively correlated with body mass index and has shown strong apoptotic and antiproliferative activities [49,50].

Breed is another factor that can influence the incidence of mammary tumors in dogs. Several studies have shown a higher incidence in pure breeds than in mixed breeds; however, there is no consensus on which breeds are at the highest risk of developing mammary tumors [14]. This information varies greatly depending on the geographical location, study type and biases. A study conducted in Spain identified Retrievers, flushing dogs and water dogs as the breeds with a higher incidence of mammary tumors [34]. In Italy, the breeds with the greatest incidence of malignant mammary tumors were Samoyeds, Dobermans, Schnauzers and Yorkshire terriers [14]. In Argentina, English Cocker Spaniels, Pekingese and German Shepherds have been reported as the breeds most frequently diagnosed with mammary tumors [31]. In Sweden, English Springer Spaniels, Dobermans and Boxers were identified as the breeds with a higher risk of developing mammary cancer [15].

A case series study performed in Mexico showed that 80% of the dogs with diagnosed mammary cancer were mixed breeds and 20% were pure breeds. Poodles and Cocker Spaniels predominated in the small breeds group, while German Shepherds, Labradors Retrievers and Rottweilers predominated in the large breeds group [5]. These results contrast with the findings reported in other countries. This case series study analyzed 1917 biopsies of mammary lesions, which are not representative of the whole canine population in Mexico because the sample was selected from biopsies sent to the Department of Pathology, Faculty of Veterinary Medicine of the National Autonomous University of Mexico, so it should be considered an estimation only [5]. Further, this data can be impacted by the low rate of dog spaying and the increased presence of street dogs in Mexico that predispose to mating between different breeds and, therefore, the population of mixed breeds might be particularly high.

### 2.2. Histological and Molecular Classification

Mammary tumors represent the most frequent neoplasia diagnosed in non-spayed female dogs, and approximately 50% are malignant [5,33,51,52]. Malignant mammary tumors have the capacity to metastasize to regional lymph nodes and to distant organs like lungs; in some cases, they can migrate through blood vessels to abdominal organs, such as the liver, spleen and kidney [51]. Over the years, several systems for the histological classification of canine mammary tumors have been established. The first classification was published in 1974 [53], the second in 1999 [54], and, subsequently, a modification was made in 2011, which is the one currently used [55].

Canine mammary tumors are highly variable in their morphology and are generally composed of more than one cell type, including luminal epithelial cells, myoepithelial cells and mesenchymal cells, in combination or alone [54,55]. They can be of epithelial origin (simple adenoma or simple carcinoma) or mesenchymal (fibroadenoma, fibrosarcoma, osteosarcoma and other sarcomas); however, some present a combination of epithelial and myoepithelial tissue (benign mixed tumors or carcinosarcoma). Mesenchymal tumors and tumors with myoepithelial cell proliferation are frequent in canines, unlike in women, where they are hardly ever diagnosed [52,56,57].

The tumor type, nuclear and cellular pleomorphism, mitotic index, presence of necrotic areas, lymphatic and peritumoral invasion and regional metastatic lymph node are some criteria used in the diagnosis of malignant mammary tumors [55]. The histological grading system in canine mammary carcinoma consists of quantifying anaplasia, tubule formation, mitotic activity and nuclear pleomorphism, as shown in Table 2.

The sum of all the individual values determines the histological grade of the malignancy (grade) [58]. The histological grade is considered to be a prognostic factor, where a higher level is associated with a poorer outcome and shorter survival rate [55,59,60,61].

In women, breast cancer tumors are classified into five molecular subtypes: luminal A, luminal B HER-2—(epidermal growth factor 2 negative), luminal B HER-2+ (epidermal growth factor 2 positive), HER-2 and triple-negative. This allows the selection of a specific targeted therapy, such as anti-estrogen drugs for the luminal A subtype, and monoclonal antibody-based immunotherapy like trastuzumab for HER-2 subtypes [62]. HER-2 is also considered an important tumor marker and is expressed in 30–35% of the canine mammary tumors [33,52]. In canine mammary cancer, multiple studies have been conducted using the same panel of markers; however, the results obtained so far, especially regarding incidence, have been highly variable and sometimes contradictory [63,64].

In a study conducted on 350 female canines with mammary carcinoma, 267 (76.3%) were classified as triple-negative, 50 (14.3%) as luminal A and 33 (9.4%) as luminal B. No HER-2-+ cases were detected [9]. Another study conducted on 73 canines identified 28 (38%) HER-2+ and 45 (52%) HER-2 cases. In that same study, 25% were luminal A, 23% luminal B, 15% HER-2+,33% basal-like subtype and 4% normal-like subtype [65]. A third study of 102 malignant mammary tumor samples classified 44.8% as luminal A, 13.5% as luminal B, 8.3% as HER-2, and 29.2% as basal type [63]. ERα is found in 50% of the benign and 22% of the malignant mammary tumors [66]. Variations in immunohistochemical assessment, small patient cohorts, different population characteristics and the geographical location of the studies could undoubtedly have contributed to these highly variable results. More studies are required, along with standardization of immunohistochemical protocols in canines and the development of dog-specific diagnostic kits. Very few anti-dog antibodies are commercially available, leading to the use of anti-human, anti-mouse or anti-rabbit antibodies, with variable specificity.

Estrogen and progesterone are essential for growth and normal mammary tissue development; however, they also influence tumor growth. Some studies have shown that the expression of the estrogen receptor (ER), progesterone receptor (PR) or both is more frequent in benign tumors and is generally associated with a favorable prognosis [67,68,69]. Another study demonstrated that estrogen receptor negative (ER-) and progesterone receptor positive (PR+) tumors correlate with a poor outcome in comparison to estrogen and progesterone positive (ER+ and PR+), while ER- and PR- tumors have the worst prognosis of all [70].

A study performed on 300 female dogs with spontaneous invasive mammary carcinoma identified triple-negative (ER/PR/HER negative) carcinoma as the most prevalent tumor type (76.3%), followed by luminal A and B (23.7%) [9]. These numbers are in contrast to the findings in humans, where in nearly 14,000 cases the predominant subtype was luminal B (43%, 5941 cases), followed by triple-negative (39%, 5402 cases), HER-2 (16%, 2271 cases) and luminal A (2%, 322 cases) [71]. In both cancer types, luminal A and B, Ki67 expression was a strong prognostic factor for outcome prediction [9]. Ki67 is a nuclear protein; as such, it can only be detected in the cell nucleus during late G1, S and G2 phases of the cell cycle and on the surface of the chromosomes during mitosis. It is among the most studied proliferation and apoptosis biomarkers in canines and is present in several tumor types. Ki67 levels are significantly lower in benign tumors than in malignant tumors, and increased levels are positively correlated with metastasis, poor prognosis and shorter survival [33,72].

E-cadherin, an adhesion membrane protein and a tumor suppressor gene, normally expressed in epithelial cells, is also an important immunohistochemical prognostic factor. Loss of E-cadherin can occur in breast cancer in women and female dogs and has been associated with increased tumor size, histological grade, stage of the disease and an overall worse prognosis [73]. In dogs, low expression or absence of E-cadherin is associated with more aggressive and metastatic cancers, including triple-negative and HER-2 positive tumors [74]. The low expression of this protein is also involved in the epithelial-mesenchymal transition, which promotes the acquisition of mesenchymal characteristics in tumor epithelial cells, invasion and cell dissemination [52,74].

### 2.3. Carcinogenesis

The tumor microenvironment is composed of the extracellular matrix (ECM), cancer stem cells (CSC), adipocytes, nerves, tumor-associated stromal cells as fibroblast and endothelial cells, infiltrating immune cells like leukocyte and macrophages and their biological products as cytokines, growth factors and molecules that contribute to tumor progression [75,76].

The extracellular matrix includes proteins that serve as a support to the tumor cells and facilitate cell–cell or cell–matrix interactions [77,78]. During the development of canine mammary tumors, the ECM suffers intense remodeling and degradation of its components and structure [78,79]. In canine mammary cancer, collagen fiber types I, III, IV and V are sparse and fibers ECM disorganized. In addition, collagen fibers are more aligned and shorter than normal tissue, which also correlates with shorter survival rates [77,78,79].

Cancer stem cells are subpopulations of tumor cells that are mainly characterized by their capacity for self-renewal and potential for differentiation and play an important role in cancer recurrence and metastasis [75,80]. Targeting cancer stem cells is used for the development of new treatments for cancer. Metastasis prognostic factors and cancer stem cell-related transcription factors that can be used to select therapeutic strategies have been identified in canine mammary tumors; these include ICAM-1, PRR14, Oct4 and Sox2 [81].

Another element that participates in the process of carcinogenesis is cancer-associated fibroblasts (CAFs). These cells are part of the stroma and participate in the epithelial-mesenchymal transition, secrete cytokines such as epidermal growth factor and transforming growth factor β and produce metalloproteinases that promote growth and tumor progression, invasiveness and metastasis [82,83,84]. In canine mammary cancer, there is an increased expression of periostin in CAFs compared to mammary adenomas, and this has a positive correlation with the histological malignancy grade [85]. Similarly, the expression of podoplanin, a protein utilized to identify CAFs, showed a positive correlation with the histological malignancy grade in canine mammary cancer [82].

The immune system plays an important dual role in cancer. It has the capacity to promote carcinogenesis but can also suppress tumor progression, depending on the subtypes of inflammatory cells, mostly lymphocytes and macrophages in the tumor microenvironment, e.g., T lymphocytes (T helper and T-FoxP3+) and macrophages subtype M2, which are in favor of tumor progression [76,86,87]. The inflammatory cells that are found in mammary tumors produce molecules, chemokines and cytokines that have proangiogenic and immunosuppressor activity. Female dogs with malignant mammary tumors that have a high level of inflammatory infiltrate, CD3+ T cells, CD4+ T cells or tumor-infiltrated macrophages have presented shorter survival times [88]. In addition, obese or overweight female dogs with malignant mammary tumors have shown a tendency for malignant tumors of higher histological grade and significantly higher levels of tumor-associated macrophages than those with normal weight, suggesting that these factors might influence the development and progression of cancer [76,86,87,88,89].

In any cell, a genetic or metabolic alteration can lead to a malignant transformation, but this is usually prevented by several molecular mechanisms that activate apoptosis. Under specific physiological conditions, DNA damage, alterations in DNA replication, poor regulation of the cell cycle, hypoxia or the accumulation of misfolded proteins, can all trigger pro-apoptotic pathways and/or anti-apoptotic suppression pathways. In cancer cells, these protective mechanisms are impaired. One of the best-described activators of apoptosis is tumor suppressor gene p53, also known as the genome guardian. In cells with severe DNA damage, the p53 gene is activated to initiate cell cycle arrest, induce a response for damage repair or trigger apoptosis if the damage is irreparable [90].

In women, p53 gene mutations have been reported in up to 30% of breast cancer cases and are generally associated with the most aggressive subtypes (e.g., triple-negative); high expression of p53 correlates with poor prognosis and shorter survival times [91]. Only a few studies have assessed p53 expression status in canine mammary cancer, and its role in progression is still unclear. In one study of 170 malignant mammary tumors in female dogs, only 0.5% (8/170) expressed p53. Tumors positive for p53 were high-grade and with high proliferative activity, suggesting that the p53 gene is involved in the progression of canine mammary cancer [92]. However, in another smaller study (40 tumor samples), a significant reduction in gene expression in eight samples, overexpression in two samples and normal expression in thirty samples was reported; a statistical analysis found no correlation between TP53 gene expression and tumor aggressiveness [93]. In another study, 7 of 35 malignant mammary tumors in dogs showed p53 immunostaining; however, the prognostic utility of p53 could not be established [94].

The neoplastic tissue is characterized by an increased cell proliferation, oncogene expression and a decreased expression of tumor suppressor genes (growth inhibitor genes). Cyclin A and cyclin D1 are protooncogenes involved in cell cycle progression that have been identified in canine mammary cancer and show a higher expression in malignant tumors. However, their usefulness as prognostic factors is limited since their expression is also particularly high in mammary dysplasia [95].

As mentioned previously, sex hormones participate in the initiation, promotion and progression of carcinogenesis of mammary tumors. Estrogen is mainly synthetized by the ovaries; however, it has been detected in high concentrations, along with some of its precursors, in malignant mammary tissue [96]. The exposure duration of mammary tissue to estrogens is key to tumor development. Benign mammary tumors and low-grade malignant tumors are usually ERα (estrogen receptor alpha) positive, while high-grade malignant tumors tend to be ERα negative by histology [67,92]. The ER1 (estrogen receptor 1) gene has a similar pattern of expression, as it is not expressed in high-grade carcinomas. Estrogen modulates gene expression and directly affects the phosphorylation (activation) of several protein kinases. As a result of these genomic and non-genomic pathways, estrogen can accelerate cell proliferation, which in turn increases the chances of acquiring new genetic errors [38].

HER-2 overexpression has been associated with poor prognosis, and HER-2 has functions in the regulation of tumor growth and cell differentiation and constitutes a marker for targeted treatment [97]. In women with breast cancer, HER-2 has been identified in 30% of the cases. In dogs, a positive correlation has been described between HER-2 expression, malignancy and high histological grade, suggesting a role in canine mammary carcinogenesis [98].

Prostaglandins (PG) are lipidic mediators involved in tumorigenic processes mainly controlled by a cyclooxygenase enzyme. PG can modulate the immune system and affect proliferative processes, apoptosis and angiogenesis [99]. Cyclooxygenases (Cox1, Cox2 and Cox3) are catalytic enzymes that are necessary for the conversion of arachidonic acid to prostaglandin G2 and subsequently to PGH2, a precursor of prostanoids (prostacyclins and thromboxanes). Isoenzyme Cox2 increases during inflammation and is implicated in the development and progression of different types of tumors, including canine mammary tumors [100,101]. Cox2 expression was associated with lymph node metastasis at the time of surgery and with the development of distant metastasis. It is also more frequent and intense in malignant (compared to benign) mammary tumors, has been reported in 56–100% of the malignant cells and is correlated with a shorter survival [101,102,103]. Cox2 modulates tumor progression through different mechanisms. In canines, elevated Cox2 expression has been associated with increased angiogenesis, proliferation and tumoral T-lymphocyte and macrophage infiltration [104,105]. In humans, these include regulation of cell proliferation, migration, invasion, secretion of growth factors by tumor cells, angiogenesis, lymph node metastasis and apoptosis [106,107,108,109].

Genetic alterations are a part of mammary tumor development. The proto-oncogene epidermal growth factor receptor (EGFR) plays an important role in human breast cancer as expression of its phosphorylated form is associated with increased angiogenesis and metastasis [110]. In malignant canine mammary carcinomas, overexpression of EGFR is associated with tumor size, necrosis, mitotic grade, histological grade of malignancy, tumor relapse, distant metastasis and clinical stage. Furthermore, it could contribute to increased angiogenesis and aggressiveness of malignant mammary tumors, suggesting that EGFR inhibitors could be used to treat metastatic disease [111,112,113].

Other common genetic alterations found in canine mammary cancer are mutations in the genes encoding proteins of the PI3K/Akt/mTOR pathway. The PI3K/Akt/mTOR pathway is necessary for the regulation of proliferation, protein synthesis, apoptosis, cell motility and angiogenesis and is dysregulated in several canine mammary tumors [57,114]. Mutations in the PIK3CA (phosphatidylinositol-4,5-bisphosphate 3-kinase catalytic subunit alpha), PTEN (phosphatase and tensin homolog), PIK3R1 (phosphoinositide-3-kinase regulatory subunit 1) and AKT1 (serine/threonine kinase 1) genes have been identified in canine mammary cancers at comparable frequencies to human breast cancers, indicating that they may be conserved across species [57]. The canine PIK3CA gene mutated in 55% and 38% of benign and malignant mammary tumors, respectively, encodes for a 1068 amino acid protein that shares 99% similarity with its human counterpart. Therefore, it is highly likely that a predisposing functional mutation of PIK3CA is comparable between humans and dogs [115]. Mutations in PIK3CA could over-activate this signaling pathway, promoting tumorigenesis [116].

Mutations in PTEN and PIK3R1 have been described in different histological types of canine mammary tumors (both benign and malignant), while AKT1 mutations were exclusively observed in complex carcinomas, suggesting that they are tissue-specific [57]. Phosphorylated AKT expression in canine mammary tumors correlates with more aggressive subtypes, lymphatic invasion and poorer survival [114]. PTEN expression correlates with less aggressive tumors in dogs [114], while its loss in canine malignant mammary tumors is associated with shorter survival [117]. Overexpression of PTEN in canine mammary tumor cells inhibits cell proliferation, induces apoptosis through upregulation of caspase-3, caspase-9 and Bax and downregulates phosphorylated AKT [118].

Mutation in the Kirsten rat sarcoma virus (KRAS) oncogene has been identified in 10.4% of canine mammary tumors (19 out of 191 cases), which is higher than reported in women with breast cancer (5%) [57]. This gene can activate the RAS signaling pathway and promote tumorigenesis. High KRAS mRNA expression in human breast cancer is associated with worse survival and an ER/PR-, HER2+ positive profile. KRAS expression is lower In the luminal A subtype and could explain the better prognosis [119]. The RAS family may be one of the main contributing factors to resistance to treatment [120].

Mutations affecting the marker of proliferation Ki-67 gene (MKI67), encoding the Ki-67 protein, have also been identified in canine mammary tumors, but at a much lower rate (6% of cases) [57]. Another important difference between human and canine breast cancer is the overexpression of ERBB2, which is exclusively found in the human breast cancer HER-2+ subtype and is not yet identified in canine mammary carcinoma [57].

Breast cancer genes 1 and 2 (BRCA1 and BRCA2) are important players in mammary tumor development in both humans and canines. They are tumor suppressor genes coding for a nuclear phosphoprotein involved in the cell cycle regulation and DNA damage response [121]. Loss of BCRA1 expression causes genetic instability that leads to other alterations like inactivation of tumor suppressor genes and activation of oncogenes, inducing tumorigenesis [122,123]. BRCA1 and BRCA2 gene mutations are inherited and increase the likelihood of developing mammary tumors from early adulthood. By the age of 80, women with BRCA1 and BRCA2 gene mutations have a cumulative risk of 72% and 69%, respectively, of developing breast cancer [124]. In canines, mutations in these genes could also predispose certain breeds to develop mammary tumors, with BRCA1 being more strongly associated with malignant cases in canines [125]. In addition, a shift of BRCA1 protein expression from exclusively nuclear to both nuclear and cytoplasmic is characteristic of canine mammary tumors, especially of the malignant type [126]. BRCA2 expression is lower in canine mammary tumors than in unaffected mammary tissues and therefore could be another possible mechanism leading to tumorigenesis [127].

As described above, although the molecular signature of canine mammary cancer is very similar to human breast cancer, it is not identical. Another example of that is the expression of special AT-rich sequence-binding protein-1 (SATB1), a global chromatin organizer and transcription factor. In canine malignant mammary tumors, SATB1 levels are decreased in both adenomas and in metastatic tumors [128]. In humans, it has been suggested to be key to promoting growth and metastasis in mammary cancer, and its overexpression is related to poor prognosis [129]. The molecular changes in canine mammary cancer described previously are included in Table 3.

### 2.4. Clinical Signs

**Figure 2:**
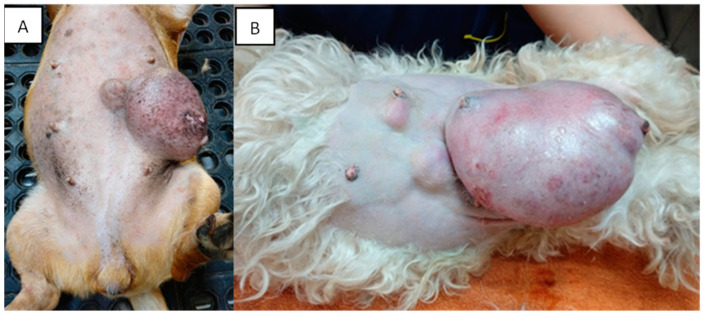
Canines with multiple mammary tumors localized in different glands. Tumor measurements larger than 5 cm in diameter with inflammation (**A**) and ulcerated skin (**A**,**B**) can be seen (own photo).

Mammary tumors are usually firm, well-defined nodules and their size can vary from millimeters to centimeters. They can occur in multiple glands at the same time and be of different histological types and grades. In addition, multiple tumors can coexist in the same mammary gland. The caudal abdominal glands are more frequently affected (up to 60% of cases) than the thoracic glands [130]. The skin in the affected area can be ulcerated or traumatized, as shown in Figure 2. Evaluation and palpation of regional lymph nodes are mandatory during diagnosis.

**Figure 3:**
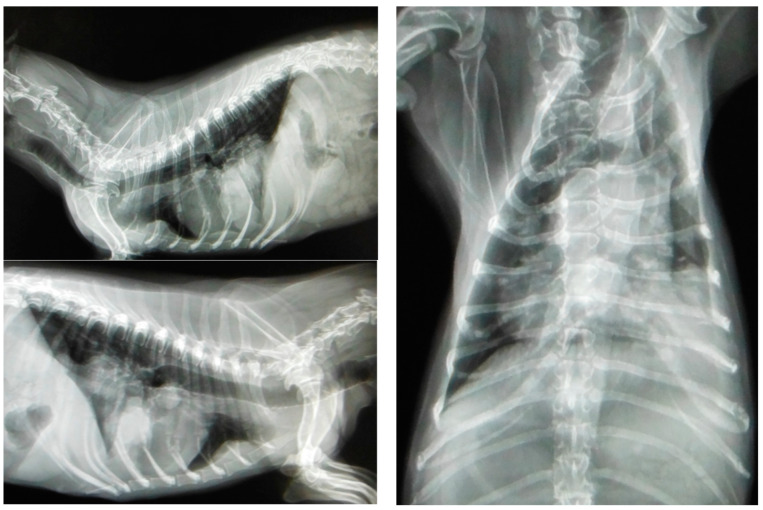
Three projections of thoracic radiographs, right and left lateral and ventrodorsal, with nodular interstitial pattern in a 12-year-old Dachshund patient with metastatic mammary carcinoma (own photo).

Most canines with mammary tumors are clinically healthy at the time of diagnosis [130]. However, patients with metastasis can present fatigue, lethargy, weight loss, dyspnea, cough, edema or lameness. Clinical signs depend on the extension and localization of metastasis [24,52]. Approximately 50% of mammary carcinomas metastasize to regional lymph nodes. Lymph node involvement is variable and can promote distant metastasis, most frequently to the lung (see Figure 3); metastatic bone lesions may also occur [24,52,131].

### 2.5. Diagnosis

Mammary tumor diagnosis is usually made either by an accidental finding during a physical exam or in patients who attend the veterinary consultation due to the presence of one or multiple nodules in the mammary glands. The definitive diagnosis and tumor grade are established based on histopathological analysis. Historically, mammary cancer classification consisted of establishing the type and histologic grade of the tumor. Nowadays, ERα, PR and HER-2 overexpression are also included, helping to better assess prognosis and establish appropriate treatment regimens in the medical practice [9].

An excisional biopsy is a good option for histopathological diagnosis of the tumor as it allows a complete histological evaluation and, in some cases, can be therapeutic. If the patient presents with multiple tumors, each tumor should be evaluated individually as different tumor types can be present in the same patient [24,132].

Fine needle aspiration (FNA) does not always lead to a diagnosis, mostly due to the heterogeneity of canine tumors and the inherent variability of cell morphology in different tumor areas. Therefore, the exact differentiation between benign and malignant tumors of epithelial origin often cannot be made. Although several studies in canines have reported low sensitivity and specificity rates of FNA cytology, Simon and collaborators demonstrated an 88% sensitivity and 96% specificity in malignant mammary tumor diagnosis by cytology (using histopathology as the gold standard). They collected at least four samples per tumor, which increased the probability of evaluating different areas of a heterogeneous tumor, and all samples were assessed by two observers [133].

Cytology can be useful to rule out differential diagnoses such as mastitis, lipomas, mast cell tumors and others. Although performing FNA of the tumor during clinical evaluation does not interfere with the surgical planning of the patients, the type of surgery is determined by the size of the lesion, the affected mammary glands and the lymphatic drainage [130].

### 2.6. Staging and Prognosis

Mammary tumors are staged using the tumor, lymph node, metastasis (TNM) system, established by the World Health Organization (WHO). According to this system, the patient is placed in one of five clinical stages based on tumor size, lymph node status and presence of metastasis. Stages I–III are assigned to non-metastatic patients (depending on their tumor size), while lymph node metastasis is classified as IV regardless of tumor size, and distant metastasis is classified as stage V, as described in Table 4 [134,135].

Staging of all patients with mammary tumors is important because mammary carcinomas can metastasize through lymphatic vessels to lymph nodes and lungs (mainly). Lymphatic drainage should be assessed, and clinical exploration of the regional lymph nodes should be made in case they are palpable or enlarged. A tissue sample should be taken for cytology or histopathology evaluation to confirm or discard metastasis [135].

Lungs are the most common site of metastasis in mammary tumors; therefore, radiographic studies of the chest in its three projections (Li-Ld, Ld-Li and VD) are essential to evaluate the presence of metastasis in the lungs. Other less frequent sites of metastasis are the liver, bone, brain, spleen, kidney, adrenal gland, uterus, heart, muscle and pancreas. Additional studies such as abdominal ultrasound, bone X-rays or CT scans are necessary to assess these possible scenarios [61,135].

Histopathological diagnosis provides information about the tumor type, histological grade of malignancy, presence of vascular or lymphatic permeation, surgery margins, lymph node status and tumor size. This information, together with the staging, helps in establishing the prognosis in canines with mammary tumors. Patients with complex carcinoma or simple tubular carcinoma have prolonged survival, unlike canines with simple tubulopapillary carcinoma, intraductal papillary carcinoma and malignant myoepithelioma that have more than a 10-fold risk of tumor-related death [59].

A retrospective case series study of 79 female dogs with malignant mammary tumors showed that patients with tumors in clinical stages IV and V, have a post-surgery survival of 6 months, unlike patients in early clinical stages (I, II or III) that survive for longer. If ovariohysterectomy is performed at the time of tumor removal, canines are more likely to live over 2 years; however, this could also be affected by the tumor type diagnosed by histopathology [136]. Another 2-year prospective study of 229 female dogs in Italy found a survival time of 18 months for canine patients diagnosed with adenosquamous carcinoma, 14 months for comedocarcinoma, 8 months for solid carcinoma and 3 months for anaplastic carcinoma and carcinosarcoma. These last two showed the highest rates of metastasis (89% and 100%, respectively) [59].

### 2.7. Treatment

The treatment of choice for mammary tumors in dogs is surgery, except for inflammatory carcinoma, where palliative medical treatment and chemotherapy are preferred [137]. The extent of surgery depends on the size and location of the tumor, as well as the presence of lymphatic drainage from the affected mammary gland [138]. Malignant tumors are significantly larger than benign ones, and 60% of patients have been reported to have multiple mammary tumors, which behave as independent primary tumors with different histopathological characteristics [139,140]. The goal of surgery is to remove all tumors with full surgical margins and/or prevent new mammary tumor formation. Canines with negative clinical or histopathological prognostic factors are not effectively treated with surgery alone and are at a higher risk of developing new mammary tumors [138,139].

An additional benefit of a surgical resection of mammary tumors is that it allows for histopathological examination of the tissue. Therefore, it has been associated with increased survival time and quality of life of patients. In addition, in some cases, it can be curative. This is especially the case for benign tumors, malignant low histological grade tumors or patients in early stages, except for inflammatory carcinoma or metastatic tumors [130].

Depending on the tumor size, location and number, surgery can be a simple mastectomy, regional mastectomy, radical mastectomy or a combination of these procedures. In patients with large tumors, lymph node metastases or unfavorable histopathological characteristics, local therapy is usually not effective and systemic treatment such as chemotherapy or hormonal therapy is required [135,141].

The lymphatic system is considered the main route of metastasis of canine mammary cancer. This is one of the reasons why the lymph node and the glands associated with lymphatic drainage are also removed during surgical excision of the mammary tumor. In healthy canines, the lymphatic vessels drain to the ipsilateral lymph nodes. While there is no drainage to the contralateral lymph node or gland, this can be altered by the presence of a mammary neoplasm [142,143].

A study of 41 cases with mammary neoplasms assessed the lymphatic drainage using indirect lymphography. The authors found that the first and second neoplastic mammary glands drain to the axillary ipsilateral lymph node and infrequently to the ipsilateral and sternal axillary simultaneously. The third mammary gland drains to the ipsilateral axillary lymph node and superficial inguinal simultaneously, but sometimes only cranially to the ipsilateral axillary lymph node. It rarely drains only caudally to the ipsilateral superficial inguinal and medial iliac lymph nodes. The fourth mammary gland drains only caudally to the ipsilateral superficial inguinal lymph node and rarely to the axillary and superficial inguinal lymph nodes simultaneously. The fifth mammary gland drains to the superficial inguinal lymph node and rarely to the ipsilateral popliteal lymph node and the lymphatic plexus medially (Table 5) [143].

Knowledge of the lymphatic drainage of the mammary glands is essential for the surgeon to determine the extent of the surgery and to better establish the post-surgical prognosis for the patient. However, the type and extent of surgery for mammary tumors is not currently standardized. Further, prospective clinical studies are required to determine how much tissue should be removed in each case and to establish whether the patient requires simple lumpectomy, local mastectomy, regional mastectomy, radical mastectomy or bilateral radical mastectomy [141]. A study assessing the evolution of canines that underwent a regional mastectomy to remove a single mammary tumor found that 58% of the patients developed a new tumor in the same chain of the mammary gland after the first surgery. Out of those, 77% underwent a second surgery. The authors conclude that an initial radical mastectomy could have prevented new mammary tumor formation and thereby helped to avoid a second surgery [144].

A randomized controlled trial of the effect of performing an ovariohysterectomy at the time of surgical removal of nonmalignant mammary tumors was conducted on 42 canines randomized to two groups. One group underwent ovariohysterectomy and mastectomy, while the other group only had mastectomy performed. Of the 42 patients in the sexually intact group, 27 (64%) developed new mammary tumors, whereas only 15 (36%) of the dogs that underwent ovariohysterectomy and mastectomy had reoccurrence of mammary neoplasms. These findings suggest that ovariohysterectomy should be performed at the time of removal of the mammary tumors to reduce the risk of developing new tumors [145].

Although surgical excision continues to be the treatment of choice for mammary carcinomas, it cannot be considered curative in canines with vascular or lymphatic invasion, due to the potential for metastatic disease at the regional or distant level. In these cases, an additional treatment option should be recommended to delay the appearance of metastasis and improve patient survival. There is no standard treatment after surgery for patients with advanced local disease, metastatic mammary carcinoma or biologically aggressive histopathological types such as solid carcinomas, micropapillary carcinomas, anaplastic carcinomas and carcinosarcomas. However, they can benefit from adjuvant treatments such as radiotherapy, non-steroidal anti-inflammatory drugs, chemotherapy, hormonal therapy or anti-angiogenic therapy, even when metastasis is not evident in the lymph node or lungs [24,130,141].

Chemotherapy as an adjuvant or palliative therapy, or in cases of metastatic disease, is routinely used in women with breast cancer and has been shown to improve survival [24]. In veterinary medicine, several chemotherapeutic protocols have been used in dogs with malignant mammary tumors, as shown in Table 6 and discussed below. However, additional prospective studies are required to verify their benefit in the survival of patients with mammary carcinoma [146]. Chemotherapy is recommended in patients at high risk of metastasis or recurrence characterized by regional lymph node metastasis, large tumors (>3 cm) and aggressive histopathological diagnosis such as high histological grade, vascular or lymphatic permeation [147]. As there is limited information on the efficacy of chemotherapy in canine patients with mammary cancer, a more in-depth assessment, including randomized controlled trials, is needed to establish guidelines for its use.

Immunohistochemical studies have demonstrated that the expression of Cox1 and Cox2 in canine mammary carcinomas, and especially elevated Cox2 expression, is associated with malignancy, a poor prognosis and tumor angiogenesis [102,138,151]. A study conducted on 28 female canines with malignant mammary tumors tested adjuvant therapy with a Cox2 inhibitor. After surgery, dogs were allocated alternatively into two treatment groups, eight dogs were treated with five doses of 5.5 mg/m^2^ mitoxantrone (chemotherapy) every 21 days, and seven patients were treated with firocoxib (Cox2 inhibitor) at 5 mg/kg/day for 24 months. The control group (*n* = 13) included dogs whose owners rejected adjuvant therapy. These patients did not receive any additional treatment. The patients were followed for 2 years or until death. Firocoxib treatment reduced the incidence of metastasis. The highest disease-free survival and overall survival was also achieved in canines that received firocoxib, with a statistically significant difference when compared to the control group. These findings support the potentially beneficial outcome of Cox2 inhibitors when used for the treatment of highly malignant canine mammary tumors [150].

Systemic hormonal treatments, including tamoxifen, a selective estrogen receptor inhibitor, are used as part of the treatment of breast cancer in women. In a study evaluating the administration of tamoxifen at 1 mg/kg orally in canines with malignant mammary tumors, 56% of the patients developed complications including pyometra, vulvar inflammation and signs of pseudopregnancy [152]. Another study evaluated the effects of tamoxifen in healthy female canines using a dose ranging from 0.5 mg/kg to 0.8 mg/kg/day. The adverse effects were similar to those previously reported: vulvar inflammation, vaginal discharge, retinitis and pyometra [153]. Due to the side effects observed in canine patients, tamoxifen is not recommended as a hormonal treatment for mammary cancer.

Immunotherapy strategies against breast cancer have been developing over time. Nevertheless, there is limited information and options for treatments based on immunotherapy in canine mammary cancer. The programmed cell death protein 1 and programmed death-ligand 1 (PD-1 and PD-L1) are expressed in mammary carcinoma and have an immunosuppressive function and inhibit cytotoxic lymphocyte T function [154]. A mouse monoclonal antibody against the PD-L1 molecule has been generated; however, further investigations are needed to prove the therapeutic effects in canines with mammary carcinoma [155].

Syndecan-1 or CD138 modulates cell proliferation, cell-matrix adhesion and migration. This protein is considered to be a poor prognostic factor and is often expressed in aggressive carcinomas. Murine monoclonal antibodies against canine CD138 were generated for nuclear medicine and preclinical trials as immuno-PET/CT or immuno-SPECT/CT and for radioimmunotherapy [156].

## 3. Challenges and Future Perspectives in the Management of Mammary Cancer in Canines

Mammary cancer is the most diagnosed malignancy in women and female dogs; in women, it is the leading cause of cancer-related death worldwide [3]. Surgery is the main treatment option for mammary tumors in canines; however, the lack of sufficient prospective randomized controlled trials has prevented the establishment of guidelines for the selection of the type and extent of surgery for each individual case, be it simple lumpectomy, local mastectomy, regional mastectomy, unilateral or bilateral radical mastectomy [141]. A few studies have reported the use of chemotherapy in dogs, including using doxorubicin, carboplatin, cyclophosphamide and 5-fluorouracil, mitoxantrone and docetaxel, with variable results and toxicities [138,148,149,150]. The criteria for selecting and implementing the type of chemotherapy are not standardized. A consensus process and more randomized controlled trials are required to establish these criteria, in addition to developing and evaluating new antineoplastic compounds that could be of benefit to canine mammary cancer patients and with fewer adverse effects.

Innovative treatments focusing on targeted and individualized molecular therapies to reduce the adverse effects of conventional chemotherapy have been developed for women with breast cancer [157]. An example of targeted therapy is the use of tyrosine kinase inhibitors such as trastuzumab, anti-HER-2 (ErbB-2) monoclonal antibody and cetuximab antibody used for the treatment of over-expressed EGFR (ErbB-1) in carcinomas and other tumors. None of these have yet been tested in canine patients.

There is a 91% amino acid homology between human and dog ErbB-1 and 92% for ErbB-2. Trastuzumab and cetuximab antibodies recognize these homologs and inhibit neoplastic growth in canine mammary cancer cell lines [158]. In another experimental study, rivoceranib, a tyrosine kinase inhibitor targeting the vascular endothelial growth factor receptor 2 (VEGFR2), induced a significant reduction in the proliferation and migration of canine mammary carcinoma cells [159]. These drugs should be further evaluated in in vivo studies and clinical trials and considered for the development of new therapies for mammary cancer in canines.

Canines are a great example of comparative oncology (that is, the use of naturally developing cancers in animals as models for the study of human neoplasms) as they develop spontaneous mammary tumors that share a high degree of similarity with human breast cancers [52]. Several molecular features of canine mammary carcinoma, including variable ER expression status, cyclooxygenase overexpression, p53 mutations, characteristics of tumor microenvironment such as the presence and role of cancer-associated fibroblast, macrophages and T-lymphocytes are very similar to those seen in humans [76,160]. The short lifespan of dogs allows for the evaluation of treatment response and to obtain results in clinical assays in a shorter timeframe. Procedures that are commonly performed in human medicine, including sample collection, surgery and imageology, are easier to execute in dogs than in murine models [52]. All these factors make canines an attractive animal model for the study of mammary cancer carcinogenesis [88,89,90].

Although several efforts to describe the molecular and histopathological characteristics of canine mammary tumors have been made, there is still a lack of agreement in the obtained results. An excellent example of that is the large variability seen in the incidence rates of molecular subtypes of mammary cancer reported by different studies [28,63,64,65,66]. There is a need for clinical studies of canine mammary cancer that use standardized procedures, canine-specific markers (e.g., antibodies) and larger and more homogenous patient cohorts to further validate the results obtained so far. The selection of cases should be strictly based on the histopathological diagnosis, and patients with previous reports of other malignant neoplasms, mammary tumor surgeries or treatments should be excluded. The inclusion of a suitable control is also recommended. Tumor size and lymph node status should be histopathologically assessed and a molecular classification established by at least two independent pathologists using canine-specific monoclonal antibodies. Lastly, during the study, spayed and non-spayed animals should be randomized into separate groups and stratified by clinical stage at the time of mastectomy to help avoid the effects of potential confounders.

Another challenge is the largely undetermined epidemiology of canine mammary cancer in many countries. Very few studies are available worldwide, and many were not performed in the same time period (in addition, some are not very recent); therefore, direct comparisons cannot be made. In many cases, the reported incidence rates may be unreliable due to the lack of a representative sample and reliable measures of dog population sizes [29]. The characterization of the canine population at risk is important for determining the magnitude of the problem and to better test and target prevention and treatment strategies.

The development of canine mammary tumors correlates with the timing of ovariohysterectomy, a procedure performed routinely in developed countries. This is one of the reasons why fewer canine mammary tumor cases are reported in countries such as the USA. Having an institution for canine registration would help to determine the canine population in each country and city, promote the development of a network where the veterinary community can register their diagnoses and have a record of all dogs that underwent pathological examination. Examples of such institutions are the Swiss Canine Cancer Registry, Danish Veterinary Cancer Register and Vet-OncoNet [15,20,23,161]. Knowing the situation in each country would help in the planning and evaluation of strategies to prevent mammary cancer and the development of guidelines for the management of canine mammary cancer.

## 4. Conclusions

Mammary cancer is one of the most frequently diagnosed malignant neoplasms in canines, and it is the most frequent tumor in non-spayed female dogs. Similarities and differences have been demonstrated between mammary cancer in women and canines at the molecular level. These could serve as a basis for a better understanding of mammary cancer pathology, the development of new therapies and diagnostic tools, the establishment of classifications and meeting the concept of the One Health approach for the benefit of both species. However, epidemiological information in canines is limited, as few countries (and cities) have managed to properly document the clinical, pathological and epidemiological characteristics of mammary cancer in canines. Promoting the publication of research into different aspects of mammary cancer, establishing a collaborative network between different countries and determining the characteristics of dog populations will favor a better understanding of the disease. In addition, both surgical and chemotherapeutic procedures need to be standardized to improve response rates and survival of mammary cancer patients.

## Figures and Tables

**Table 1:** Canine mammary cancer epidemiology.

Country (City/State)	Incidence	Year [Reference]
Italy	193 per 100,000	2001–2008 [13]
Italy (Venice)	250 per 100,000	2005–2013 [14]
Sweden	111 per 10,000	1995–2002 [15]
United Kingdom	205 per 100,000	1997–1998 [16]
Italy (Genoa)	181.8 per 100,000	2000–2002 [17]
Italy (Genoa)	196.6 per 100,00	1995–1999 [17]
Italy (Genoa)	264 per 100,000	1990–1994 [17]
Italy (Genoa)	119.2 per 100,000	1985–1989 [17]
USA (California)	145 per 100,000	1963–1968 [18]

**Table 2:** Criteria used for the histologic grading of malignancy in canine mammary carcinomas [55,58].

Tubule Formation	Nuclear Pleomorphism	Mitosis
Tubule formation >75%: 1 point	Uniform or regular small nucleus and occasional nucleoli: 1 point	0–9: 1 point
Moderate formation of tubular arrangement (10–75%) admixed with areas of solid tumor: 2 points	Moderate degree of variation in nuclear size and shape, hyperchromatic nucleus (some of which can be prominent): 2 points	10–19: 2 points
Minimal or no tubule formation (<10%): 3 points	Marked variation in nuclear size and hyperchromatic nucleus, often with one or more prominent nucleoli: 3 points	>20: 3 points
**Total Score**	**Grade of Malignancy**	
3–5	I (low) well differentiated	
6–7	II (intermediate) moderately differentiated	
8–9	III (high) poorly differentiated	

**Table 3:** Molecular alterations in canine mammary tumors.

Molecular Feature	Percentage of Affected Cases/Observations	Number of Cases and Type(s) of Mammary Tumor(S)	Model	Country	Reference
eRα expression	7.76% (adenocarcinomas), 4.46% (tumors without tubular formation), 36% (benign tumors)	28 malignant and 43 benign tumors	Tissue	Spain	[67]
HER-2 expression	53.3%	15 mammary tumors	Tissue	Brazil	[98]
*BRCA1* and *BRCA2* expression	BRCA1: 97% (91% in controls); BRCA2: 97% (88% in controls)	100 mammary tumors	Tissue	Sweden	[125]
*SATB1* expression	Reduced expression in metastases. No significant expression differences between adenomas and carcinomas.	13 carcinomas with lymph node metastasis and 10 simple adenomas	Tissue	Germany	[128]
EGFR overexpression	55.7%	61 mammary carcinomas	Tissue	Portugal	[111]
*PTEN* overexpression	Inhibits cell proliferation	Canine mammary tumor (PTEN transfected CHMp and CHMm cells)	Cell lines	Japan	[118]
Cyclin D1 overexpression	60% (pre-cancerous), 40% (cancerous)	28 pre-cancerous and cancerous lesions	Tissue	Italy	[95]
p53 mutation	0.5%	170 mammary carcinomas	Tissue	Italy	[92]
*PIK3CA* mutation	55% (benign) and 38% (malignant)	183 canine mammary tumors, 40 benign and 143 malignant	Tissue/ Genes	Republic of Korea	[115]
*AKT1* mutation	0% (benign) and 9% (malignant complex carcinomas). 0/78 vs. 8/44 in simple and complex carcinomas, respectively	183 canine mammary tumors, 40 benign and 143 malignant	Tissue/ Genes	Republic of Korea	[57]
*TP53* mutation	0% benign and 15% malignant	183 canine mammary tumors, 40 benign and 143 malignant	Tissue/ Genes	Republic of Korea	[57]
*PTEN* mutation	4% benign and 20% malignant	183 canine mammary tumors, 40 benign and 143 malignant	Tissue/ Genes	Republic of Korea	[57]
*PIK3RI* mutation	2% benign and 10% malignant	183 canine mammary tumors, 40 benign and 143 malignant	Tissue/ Genes	Republic of Korea	[57]
*KRAS* mutation	19 cases (10.4%)	183 canine mammary tumors	Tissue/ Genes	Republic of Korea	[57]

**Table 4:** Staging of canine mammary tumors using the tumor, lymph node, metastasis system [134].

T: Tumor size
T1 < 3 cm diameter
T2 3–5 cm diameter
T3 > 5cm diameter
N: lymph node status
N0: without histological or cytological metastasis
N1: with histological or cytological metastasis
M: distant metastasis
M0: without distant metastasis
M1: with distant metastasis
Clinical stages
I T1 N0 M0
II T2 N0 M0
III T3 N0 M0
IV any T with N1 and M0
V any T with N1 and M1

**Table 5:** Lymphatic drainage of normal and neoplastic canine mammary glands [143].

Mammary Gland	Normal Lymphatic Drainage	Neoplastic Lymphatic Drainage
1.Cranial thoracic	LN axillary	LN axillary and sternal
2. Caudal thoracic	LN axillary	LN axillar and sternal
3. Cranial abdominal	LN axillary and inguinal	LN axillary, inguinal and medial iliac
4. Caudal abdominal	LN inguinal	LN inguinal and axillary
5. Inguinal	LN inguinal	LN inguinal, popliteal

**Table 6:** Chemotherapy protocols for canine mammary cancer.

Drugs	Dose	Number of Patients	Results	Reference
5-Fluorouracil and cyclophosphamide	150 mg/m^2^ and 100 mg/m^2^	16	Group 1—71.4% died of metastasis within the first 2 years	[148]
		Group 1 (8 surgery)	Group 2—100% lived longer than 2 years.	
		Group 2 (8 surgery + chemotherapy)	Significant difference in overall survival time: 24 months (Group 2), 6 months (Group 1)	
			Toxicity: temporary leukopenia associated with chemotherapy in group 2	
Doxorubicin or docetaxel	30 mg/m^2^ or 30 mg/m^2^	31	Patients with only surgery showed a survival of 390 days and patients treated with surgery + chemotherapy 231 days.	[149]
		Group 1 (19 surgery)	No significant difference in the recurrence-free interval, time to metastasis, and overall survival	
		Group 2 (12 surgery + chemotherapy)	Toxicity: mild allergic skin reactions with docetaxel	
Carboplatin and piroxicam/firocoxib	300 mg/m^2^	29	Survival of the group treated with only surgery was 63 days.	[138]
	0.3 mg/kg/day/	Group 1 (7 surgery), Group 2 (8 surgery + carboplatin)	Survival of group treated with surgery + chemotherapy did not reach the median survival.	
	5/mg/kg/day	Group 3 (5 surgery + carboplatin + piroxicam),	Survival of group treated with surgery + chemotherapy + piroxicam was 390 days.	
		Group 4 (9 surgery + carboplatin + firocoxib)	Survival of group treated with surgery + chemotherapy + firocoxib was 570 days.	
			Increased median survival in piroxicam, and firocoxib groups (390 and 570 days respectively vs. 63 days surgery only)	
			Toxicity: 1 death for adverse effects of piroxicam in group 3	
Mitoxantrone and firocoxib	5.5 mg/m^2^ and 5/mg/kg/day	28	Patients treated with only surgery had a survival of 12.7 ± 0.8 months.	[150]
		Group 1 (13 surgery),	Patients treated with surgery + chemotherapy had a survival of 16.5 ± 2.6 months.	
		Group 2 (8 surgery + mitoxantrone),	Patients treated with surgery + chemotherapy + firocoxib had a survival of 19.4 ± 2.1 months.	
		Group 3 (7 surgery + firocoxib)	Significantly higher disease-free survival in surgery + mitoxantrone and surgery + firocoxib groups than in control (surgery only)	
			Toxicity: 1 dog neutropenia grade 3 and gastrointestinal toxicity grade 2 from group 1. In group 2, 2 dogs urea and creatinine increased, grade 1 and 2	
